# Ewen D. Cameron: Wechselwirkungen zwischen einem forschenden Psychiater und seinen professionellen wie auch gesellschaftlichen Kontexten

**DOI:** 10.1007/s00115-022-01269-3

**Published:** 2022-02-21

**Authors:** Hanfried Helmchen

**Affiliations:** Klinik für Psychiatrie und Psychotherapie, CBF, Charité – Universitätsmedizin Berlin, Hindenburgdamm 30, 12200 Berlin, Deutschland

**Keywords:** Klinischer Kontext, Gesellschaftlicher Kontext, Psychiatrische Somatotherapie, CIA, Bioethik, Clinical context, Societal context, Psychiatric somatotherapy (depatterning Psychic driving), CIA, Bioethics

## Abstract

Der Psychiater Ewen Cameron hat bereits in den 1930er Jahren organisatorische Grundlagen für die sozialpsychiatrische Reform der psychiatrischen „Anstalt“ gelegt, das open-door-System eingeführt und 1946 die weltweit erste psychiatrische Tagesklinik gegründet. Ebenso hat er mit dem „psychic driving“ eine „automatisierte Psychotherapie“ entwickelt sowie die somatischen Behandlungsverfahren seiner Zeit (wissenschaftlicher Kontext) zu einer neuen Therapieform gebündelt, dem „depatterning“. Sein Bild in der Öffentlichkeit wurde zunächst durch seine charismatische und mit bedeutenden Ehrungen anerkannte Persönlichkeit geprägt, u.a. wurde Cameron 1961 Gründungspräsident des Weltverbandes für Psychiatrie (WPA). Als 1977 bekannt wurde, dass eines seiner Forschungsprojekte von der CIA verdeckt mitfinanziert worden war, wurde er zum Inbegriff des brainwashing-Experten stilisiert. Die in die gesellschaftliche Atmosphäre des kalten Krieges mit ihren Verschwörungsvermutungen eingebettete öffentliche Beschäftigung mit der „Gehirnwäsche“ und den Gedankenkontrolltechniken der CIA (gesellschaftlicher Kontext) veränderte das posthume Bild von Ewen Cameron grundlegend. Im kritischen Rückblick hat er seine Forschung in einem klinischen Kontext realisieren können, das sowohl sein Forschungskonzept tolerierte und offenbar höher als das Leiden des einzelnen Kranken bewertete, als auch die klaren ethischen Regeln des Jahres 1948 (Nürnberger Kodex, Genfer Deklaration des Weltärztebundes wie auch der UN-Deklaration der Allgemeinen Menschenrechte) noch nicht internalisiert hatte. Auch mit seinen sozialtechnischen Phantasien lief Cameron Gefahr, die sozialpsychiatrische Versorgung psychisch Kranker zu verlassen und in eine totalitäre Ideologie abzugleiten.

## Abstract (en)

As early as in the 1930s the psychiatrist Ewen Cameron laid the organisational fundamentals for the reform of psychiatric hospitals, introduced the open-door system, and founded the worldwide first psychiatric day-hospital in 1946. He also developed an automated psychotherapy, called “psychic driving“; furthermore, he bundled the somatic treatments of his time (scientific context) to a new form of treatment that he called “depatterning”. His public image initially was determined by his charismatic personality, which received important honors; e.g. in 1961 he became the founding president of the World Psychiatric Association (WPA). Ten years after his death in 1967, when it became known that one of his research projects was covertly cofinanced by the CIA, he was stylized as the incarnation of brainwashing. The public concern with “brain-washing” and the mind control techniques of the CIA embedded in the societal atmosphere of the “cold war” with its conspiracy conjectures (societal context) changed fundamentally the posthumous image of Cameron. In a critical look back the fact remains that Cameron could have realized his research concepts in a clinical context that tolerated his research against the disease and emphasized this as opposed to the immediate suffering of the individual patient and, at the same time did not completely internalize the clear ethical regulations of the year 1948: the Nuremberg Codex, the World Medical Association‘s- Declaration of Geneva as well as the UN-Declaration of Human Rights. Furthermore, in his socially technical phantasies Cameron was in danger of leaving the socially oriented psychiatric care of mentally ill patients and of slipping down into a totalitarian ideology.

History is too important to become mere hagiography [1]

Heute werden wir angehalten, uns schrecklicher Taten der Vergangenheit zu erinnern, um ihre Wiederholung zu vermeiden. Wenn aber diese Taten aus heutiger Sicht so furchtbar sind, dann stellt sich die Frage, warum sie in der Epoche, in der sie begangen wurden, anscheinend als nicht so schrecklich angesehen wurden, zumindest nicht als so schrecklich, dass öffentlicher Protest sie blockiert hätte. Es geht also um die Frage, in welchem Kontext die Taten stattfanden. Wirkungen des Kontextes werden fassbar, wenn Menschen, die wegen ihrer Leistungen in der Öffentlichkeit ihrer Zeit anerkannt waren, wegen derselben oder anderer Leistungen in einem zeitlich wie meist auch gesellschaftlich anderen Kontext aber verurteilt wurden; entweder war erst dann eine Schattenseite ihrer Leistung oder ihrer Persönlichkeit bekannt geworden oder aber die Maßstäbe zur Beurteilung ihrer Leistung hatten sich erheblich oder gar grundlegend geändert. Dies sei mit der folgenden Skizze illustriert, die verschiedene Kontexte der dunklen Seite des im hellsten Licht fachlicher Anerkennung erscheinenden Psychiaters Donald Ewen Cameron deutlich zu machen sucht, und zwar in zweifacher Hinsicht: im Einfluss der klinischen Praxis seiner Zeit auf die Behandlung seiner Patienten und in der Wirkung seiner psychiatrischen Praxis auf sein soziales Umfeld. Es geht um die Wechselwirkungen zwischen einer charismatischen Persönlichkeit und ihrem psychiatrischen Umfeld: Es ließ zu, dass Cameron mit seiner Konzeption einer neuartigen Therapie eher den Erfolg eines therapeutischen Durchbruchs suchen konnte als das individuelle Leid seiner Patienten im Blick zu haben. Deutlich wird dies aus einer späteren Perspektive, in der im gesellschaftlichen Umbruch ab den 1960er-Jahren die Psychiatrie den einzelnen Patienten ernst zu nehmen beginnt. Aber auch in umgekehrter Richtung werden Gefahren wie die eines ideologischen Abgleitens ins Totalitäre deutlich, wenn Cameron versucht, mit einem eigenen Konzept von Sozialpsychiatrie die Gesellschaft grundlegend zu verändern und damit die mit der Behandlung psychisch kranker Individuen gesetzten Grenzen der Psychiatrie überschreitet.

## Cameron

Ewen D. Cameron wurde am 24.12.1901 in Schottland geboren, graduierte 1924 an der Universität Glasgow, 1925 an der Universität London, erhielt seine psychiatrische Ausbildung bei Adolf Meyer in Baltimore und am Burghölzli in Zürich. 1929 ging er für 7 Jahre nach Brandon in der kanadischen Provinz Manitoba; von der dortigen psychiatrischen Anstalt aus organisierte er ein Netz von 10 psychiatrischen Kliniken, aus denen später gemeindepsychiatrische Zentren hervorgingen. In Brandon beschäftigte er sich auch mit der Messung von Verhaltensänderungen und schrieb ein Lehrbuch über experimentelle Psychiatrie [2]. 1936 promovierte er mit Auszeichnung in Glasgow zum M.D. und wurde im gleichen Jahr Forschungsdirektor der Neuroendocrine Research Foundation am Worcester State Hospital in Massachusetts; dort untersuchte er die kurz zuvor von Manfred Sakel beschriebene Insulin-Koma-Therapie. 1938 wurde er Professor für Psychiatrie und Neurologie an der Albany Medical School. 1943 wechselte er an die McGill University in Montreal. Als Direktor der Psychiatrischen Abteilung des Universitätsklinikums Royal Victoria Hospital führte er das „Open-door-System“ ein und gründete 1946 mit dem Allan Memorial Institute die weltweit erste psychiatrische Tagesklinik [3]. Dieses Institut entwickelte er zu einem weltbekannten psychiatrischen Ausbildungs- und Forschungszentrum, indem er diverse Forschungsgruppen mit dem Ziel etablierte, „... the unresting determination of its members to break through the old limits, to pass beyond the already known to wider and wider fields of knowledge; the regard for the dignity and work of every human being who turns to the Institute in his hour of need“ [4]. In den 1950er-Jahren wurde er zum ersten Lehrstuhlinhaber für Psychiatrie an der McGill-Universität berufen und in viele ehrenvolle Leitungsfunktionen gewählt, u. a. 1952/53 zum Präsidenten der American Psychiatric Association und 1961 zum Gründungspräsidenten der World Psychiatric Association (1961–1966). Seine Arbeit wurde durch Preise und Ehrenmitgliedschaften gewürdigt, so wurde er u. a. 1966 in Montreal für seinen „überragenden Beitrag zur psychischen Gesundheit der Kanadier“ geehrt [4]. Sein Name gehörte in diesen Jahren zu den „drei oder vier großen Namen in der Welt“. „Niemand hätte negiert, dass Cameron ein großer Mann sei“ [5].

Cameron beeindruckte als ein therapeutisch wie wissenschaftlich ungemein engagierter, kenntnisreicher, vielseitig anregender Psychiater, der seine Gedanken klar und flüssig ausdrücken sowie Mitarbeiter begeistern konnte [4]. Einige beschrieben ihn als eine unabhängige, kreative, zielgerichtete, gut organisierte Persönlichkeit mit außerordentlichen administrativen Fähigkeiten, die entschieden und mutig verfolgte, wovon auch immer sie überzeugt war [5, 6]. „It was apparent that he was vitally concerned with the well-being of men, regardless of national barriers, race, or religion“ [7]; „he hated mental illness … he could not not treat … had a furor therapeuticus“ [8]. Cameron arbeitete hart, beobachtete genau und die Befunde, die er bei seinen Patienten erhob, diktierte er präzise und detailliert unmittelbar danach in Gegenwart seiner Mitarbeiter [5]. Er war ein Mann mit Wärme und Strenge [7] und großer Ausstrahlung: Bei seinen Visiten wurde hinter ihm getuschelt „There but for the grace of God goes God“ [9]. Aber er war auch ein Getriebener, ungeduldig, suchte Abkürzungen, versuchte den psychotherapeutischen Prozess zu beschleunigen; er griff neue Ansätze schnell auf, ging aber eher in die Breite als in die Tiefe, sodass seine Forschungsergebnisse als oberflächlich angesehen wurden und nicht überzeugten [7].

Im August 1964 wechselte er als Forschungsprofessor der Medical School zurück nach Albany. Beim Bergsteigen mit seinem Sohn starb er 1967.

Warum aber verließ er das von ihm in über 20 Jahren aufgebaute psychiatrische Zentrum der McGill-Universität „fluchtartig“, fast „vertrieben“, still und ohne, dass seine Leistung gewürdigt wurde? Selbst der ausführlichen und kritischen Charakteristik Camerons durch seinen Nachfolger Cleghorn sind keine eindeutigen Gründe für diesen Ortswechsel zu entnehmen [7]. Es finden sich allenfalls Vermutungen [10] und verhaltene kritische Bemerkungen zu seinen Behandlungsmethoden, insbesondere dem „depatterning“, das Sir Aubry Lewis 1957 privatim als „barbarisch“ charakterisierte [7].

Cameron versuchte, spätestens ab 1952 bei Patienten mit therapieresistenten Zwangs- oder Wahngedanken alle Erinnerungen an die pathologischen Denkinhalte mittels hochfrequenter („regressiver“) Elektrokrampfbehandlung („depatterning“) zu löschen oder sie zumindest mit psychotomimetischen Substanzen (LSD) und sensorischer Deprivation aufzulösen („disorganizing“) und dann bei den auf diese Weise suggestibel gemachten Patienten das („leere“) Gedächtnis mit sozial verträglichen Überzeugungen wieder aufzubauen („re-programming“; [11]); dazu setzte er die Patienten kurzen Aussagen aus, die sie selbst während psychotherapeutischer Gespräche gemacht hatten. Die Patienten konnten den in andauernder monotoner Wiederholung als „Endlosschleife“ vom Tonband für Stunden oder Tage unter medikamentös induziertem Dämmerschlaf und in Kombination mit sensorischer Deprivation angebotenen Formeln nicht entgehen; Cameron beschreibt detailliert seine Technik als „psychic driving“, um damit den Widerstand der Patienten zu überwinden, bisher unzugängliches Material freizulegen und ein „dynamisches Implantat“ aufzubauen, aber auch, wie unwohl sich Patienten unter diesem Prozedere fühlten oder gar aus der Behandlung flüchteten. Die Wirksamkeit dieses Therapieverfahrens wurde unter verschiedenen Modifikationen untersucht [12]. Cameron war dazu durch die neurophysiologische Hypothese des Psychologen Donald Hebb angeregt worden, wonach neuronale Netze, die sich durch häufige Wiederholung eines Reizes bilden, Lernvorgängen zugrunde liegen [13]. Mit dem „psychic driving“ sah Cameron sich als Inventor einer neuartigen Psychotherapie, einer „automatisierten Psychotherapie“ [14].

## Der klinische Kontext

Dies fand in einem *klinisch-psychiatrischen Umfeld* statt, in dem die Komponenten des „depatterning“ auch von anderen Psychiatern eingesetzt wurden, z. B. als „regressive ECT“ oder „annihilation therapy“ [15]. Damit sollte ein hirnorganisches Syndrom mit Desorientiertheit und Gedächtnisstörungen als das wirksame Prinzip zur Auflösung psychopathologischer Muster [16] oder ein „Nichthaben-können“ psychotischer Akte [17] hervorgerufen werden. Aber Cameron intensivierte diese Wirkungen noch durch bis dahin nicht gekannte Häufigkeit und Dichte der EKT (2- bis 3‑mal täglich für 15 bis 30 Tage; [18]) sowie durch Kombination mit der von dem Schweizer Psychiater Jakob Klaesi bereits 1920 mit dem Barbiturat Somnifen eingeführten „Schlafkur“ [19] und der kurz zuvor von Donald Hebb beschriebenen sensorischen Deprivation mit psychotomimetischen Substanzen wie LSD, das bereits als psycholytische Unterstützung von Psychotherapie bekannt war [20, 21].

Der *wissenschaftlich-psychiatrische Kontext *nach dem 2. Weltkrieg war bestimmt durch eine therapeutische Aufbruchsstimmung: Die antipsychotische Wirksamkeit der sog. „Schockbehandlungsverfahren“ wie auch neue Arzneimittel ab den 1950er-Jahren erwiesen sich als spezifisch wirksam gegen psychotische Erkrankungen. Diese Verfahren wurden mit therapeutischer Zielsetzung angewandt; die spezifischen Erfahrungen mit ihren neuartigen Wirkungen wie auch unerwünschten Wirkungen wurden zunächst kasuistisch beobachtet, beschrieben, empirisch modifiziert und schließlich auch wissenschaftlich kontrolliert zum Erkenntnisgewinn eingesetzt. Zwischen therapeutischer und erkenntnissuchender (forschender, experimenteller) Intervention wurde oft nicht getrennt; es war (noch) nicht üblich, die Patienten über den Versuchscharakter der Behandlung aufzuklären und danach ihre Einwilligung einzuholen.^1^ Erst die kritische Reflektion dieser wissenschaftlich innovativen Praxis führte im letzten Drittel des letzten Jahrhunderts zu ihrer normativen Einhegung, indem Wissenschaftler und die Legislative Standards ethischer Zulässigkeit entwickelten und Ethikkommissionen zur Beratung und Kontrolle klinischer Forschung institutionalisiert wurden [22].

Die Feststellung, dass Camerons Forschungsprojekte heute von jeder Ethikkommission abgelehnt worden wären [9], bestätigt, dass die heutigen Normen klinischer Forschung zu Camerons Zeit eben noch nicht sehr entwickelt waren und noch weniger in der Forschungspraxis berücksichtigt wurden. Deutlich ist damit aber ebenfalls, dass das jahrtausendalte hippokratische Gebot, jegliche den Patienten möglicherweise schädigende Intervention zu vermeiden, Cameron zumindest zu expliziten Nutzen-Risiko-Erwägungen hätte veranlassen müssen – wie dies Claude Bernard bereits 1865 gefordert hatte [23].^2^ Stattdessen hat er eine in ihrer Intensität unübliche und damit neuartige Behandlungsmethode trotz ihrer potenziellen Schädlichkeit (Gedächtnisstörungen, psychische Traumatisierung) angewandt, ohne die zu seiner Zeit geltenden ethischen Normen zu beachten – obwohl sie ihm als forschenden und leitenden Psychiater einer weltweit führenden Institution bekannt sein mussten. Öffentliche Kritik und Ablehnung erfuhr Cameron – wenn überhaupt ([9] S. 120) – von Psychoanalytikern, die seine Behandlungsmethoden als mechanistisch und biologistisch ablehnten. Die unzureichende Kontrolle durch die wissenschaftliche Community ist jedoch nicht damit zu erklären, dass Camerons Projekte dem für alle CIA-finanzierte Forschung geltenden Gebot der Geheimhaltung unterlegen hätten – denn er hatte ja seine Methoden publiziert; immerhin wurden sie niemals repliziert [9]. Doch scheint die unzureichende Kontrolle zum einen aus dem wissenschaftlichen Kontext zu kommen, der in jenen Jahren durch die Offenheit eines hoffnungsvollen Aufbruchs mit erstmals wirksamen Behandlungsmethoden (Elektrokrampftherapie, psychotrope Medikation) gegen chronisch psychotische Erkrankungen bestimmt war; zum anderen mag Camerons Standing als psychiatriepolitisch anerkannte und einflussreiche Persönlichkeit eine wirksame Kontrolle verhindert haben; wohl nicht zuletzt wird die fehlende Kontrolle in unzureichender Kenntnis des sich gerade erst konkret entwickelnden normativen Kontextes psychiatrischer Forschung gelegen haben.

Erstmals nachhaltigere, wenn auch nur zögerlich einsetzende Aufmerksamkeit erregten die Regeln zur Forschung mit Menschen, die im Nürnberger Ärzteprozess 1947 durch die grausamen medizinischen Experimente nationalsozialistischer Ärzte provoziert worden waren und als Nürnberger Kodex bekannt wurden [25].^3^ Die ebenfalls 1948 veröffentlichte Genfer Deklaration des Weltärztebundes (WMA) blieb jedoch weitgehend unbekannt. Erst mit der rapiden Ausweitung der klinischen Forschung seit Mitte des 20. Jahrhunderts wurde die Einwilligung nach Aufklärung in der Deklaration von Helsinki 1964 kodifiziert, 1966 mit einer Publikation von Beecher [26]^4^ öffentlich bekannt und gewann seit 1972 in den USA als Konzept des „informed consent“ erhebliche Bedeutung in der klinischen Forschung, nachfolgend auch in der klinischen Praxis [28].

## Der gesellschaftliche Kontext

Im Jahr 1977, 10 Jahre nach Camerons Tod, wurde in einer Ausschusssitzung des US-amerikanischen Senats [29] bekannt, dass Cameron Forschungsmittel auch von der CIA erhalten hatte. Danach brach ein Scherbengericht über ihn herein „as if the simple mark of ‘CIA’, once revealed, transformed the very medical, social, and personal reality of events“ [9]. Cameron wurde als „Monster“ bezeichnet. Seine Behandlungsmethoden wurden jetzt „barbarisch“ genannt; Patienten, die Cameron bewundert und sich seinen Behandlungen gefügt hatten, beschrieben sie nun jedoch als unmenschliche Tortur mit anhaltenden Spätfolgen. „My mother thought Cameron was God, he could do no wrong,“ [9] sagte Leslie Orlikow, Tochter einer der bekanntesten Patientinnen von Cameron; Collins interpretiert dies als Ausdruck einer Übertragung [8]. Einige klagten mit Erfolg gegen die CIA bzw. die Regierung. Kritisiert wurde nun auch, dass die Patienten weder aufgeklärt worden waren noch eingewilligt hatten. In der Öffentlichkeit wurde Cameron als „brainwashing expert“ gebrandmarkt.

Es mag als Ironie der Geschichte erscheinen, dass die von Cameron gesehene Bedeutung des sozialen Kontextes katastrophale Folgen für ihn selbst bzw. sein posthumes Bild hatte. Die allgemeine *soziokulturelle Atmosphäre* des „kalten Krieges“, das darin eingebettete Thema des „brainwashing“ [30] und schließlich das öffentliche Bild der CIA können als Kontexteinflüsse hier nur angedeutet werden.

In den 1950er-Jahren herrschte in den USA eine paranoide Atmosphäre: der *„kalte Krieg“* zwischen der kommunistischen Sowjetunion und den kapitalistischen USA erzeugte in den USA eine Furcht vor kommunistischer Infiltration; Parlament und Regierung bildeten Untersuchungskommissionen gegen „unamerikanische Umtriebe“ („McCarthyism“ [31]), die schließlich zu Verschwörungstheorien und einer antikommunistischen „Hexenjagd“ [32] vornehmlich auf Künstler, Intellektuelle und Politiker führten, die als Kommunisten verdächtigt wurden. Sie mussten sich von Kommissionen befragen lassen und wurden beruflich ausgegrenzt [25].

In diesem allgemeinen Kontext erlangte das Thema „brainwashing“ [30] öffentliche Resonanz [9], als nach dem Koreakrieg (1951–1953) amerikanische Soldaten aus chinesischer Gefangenschaft mit kommunistischen Überzeugungen zurückkehrten und der kommunistische Schauprozess 1949 gegen den ungarischen Kardinal Mindzenty auf ein erzwungenes Geständnis hinwies. Die Fremdkontrolle des Denkens („mind control“, „brainwashing“) wurde zu einem öffentlichen Thema. Ein kommunistischer Verhörer, der den amerikanischen Geschäftsmann Robert Vogeler zu einem solchen falschen Geständnis vor einem Budapester Gericht brachte, prahlte 1951: „If God Himself was sitting in that chair, we would make him say what we wanted him to say“ [30]. Diese Verhöre nutzten Wissen, das der russische Physiologe Iwan Pawlow durch seine Untersuchungen zu bedingten Reflexen gewonnen hatte [33].

Zur Abwehr solcher kommunistischen Gedankenkontrolltechniken finanzierte der amerikanische Geheimdienst CIA ab 1953 im Rahmen des Projekts „MK-ultra“ ein geheimes Forschungsprogramm, das auf Techniken zur Verhaltensmodifikation, genauer auf Verhörtechniken zielte, von denen einige von psychischer Folter nicht mehr zu unterscheiden waren. Es umfasste mindestens 149 Teilprojekte, u. a. Forschungsprojekte etlicher amerikanischer Psychologen, Soziologen und Psychiater, so auch Untersuchungen zum „psychic driving“ und „depatterning“ von Ewen Cameron. Es blieb unklar, ob Cameron (wie auch andere Wissenschaftler) über diese Quelle einiger seiner Forschungsmittel Bescheid wusste, da diese staatliche Finanzierung verdeckt über die Society for Investigation of Human Ecology erfolgte [34]. Kollegen Camerons meinten, dass er dieselben Untersuchungen im Verfolg seiner eigenen Forschungskonzeption auch ohne CIA-Unterstützung durchgeführt hätte. Seine als geheime CIA-Auftragsforschung beschriebenen Behandlungsmethoden des „depatterning“ und des „psychic driving“ wurden nicht geheim durchgeführt, sondern er wandte sie offen in Gegenwart seiner Mitarbeiter an: „everything Cameron did was done in the open and with knowledge of his peers“ [5]. Er publizierte sie detailliert einschließlich negativer Befunde und Ablehnung durch Patienten [11, 12, 14]. Seine Methoden wurden nun jedoch viel kritischer gesehen, da ihr Nutzen fraglich schien, ihre schädlichen Wirkungen, speziell das passagere hirnorganische Psychosyndrom des „depatterning“, jedoch nicht zu übersehen waren [5]. Schließlich wurde Cameron in diesem Prozess öffentlicher Verdammung sowie damit verbundener Verschwörungstheorien als „brainwashing avatar“ interpretiert, „der das tut, was sein Meister hinter ihm wünscht“ [9]. Die vielfältige Reaktion auf die Tatsache geheimer Finanzierung lässt offen, wieweit die betroffenen Forscher im Bereich verhaltenswissenschaftlicher Gedankenkontrolltechniken tatsächlich an ihrer spezifischen Anwendung als inhumane Verhörtechniken beteiligt waren.^5^

Aber nicht nur in der Öffentlichkeit provozierte Cameron ein widersprüchliches Bild: vom weltbekannten innovativen Psychiater zum Monster, das seine Patienten für Experimente im Auftrage eines Geheimdienstes missbraucht [9]. Auch seine wissenschaftlichen Vorstellungen waren widersprüchlich.

## Sozialpsychiatrie und verhaltenswissenschaftliche Ideologie

So sah er die alten psychiatrischen „Anstalten“ als Orte, in deren verderblich monotonen und restriktiven Einrichtungen die Persönlichkeit der Patienten zugrunde ging [9]. Wenn Patienten in solcher Umgebung Hoffnungslosigkeit lernten, dann könnte – so seine im Kontext der Verhaltensforschung lerntheoretisch unterlegte Annahme – eine offene und zwangslose Umgebung solchen negativen Verlauf abschwächen oder vermeiden. Dementsprechend öffnete er die Türen, legte bereits in den 1930er-Jahren mit der Neuordnung der psychiatrischen Versorgung in Manitoba die Grundlage für gemeindepsychiatrische Zentren, entwickelte am 1943 gegründeten Allan Memorial Institut in Montreal die weltweit erste psychiatrische Tagesklinik und forderte die langfristige Nachsorge der aus psychiatrischen Kliniken entlassenen Patienten mittels Übergangseinrichtungen [35].

Damit nicht kompatibel erscheint Camerons 1946 publiziertes Verständnis von Sozialpsychiatrie [36]. Denn er meinte, die krankhaften Ideen psychisch Kranker würden die Gesellschaft infizieren und über ihre Nachkommen weitergegeben, weshalb psychisch Kranke zu isolieren seien und an der Weitergabe ihrer krankhaften Einstellungen und Ideen an ihre Kinder gehindert werden müssten [37]. Vor dem Hintergrund seiner Forschung zu Verhaltensänderungen meinte er, dass die deutsche Bevölkerung xenophobe und in tiefsitzender Angst wurzelnde aggressive Tendenzen habe, die zur Wiederholung der Katastrophe führen würden, wenn sie nicht behandelt würden. Die Aggressivität resultiere aus Schwäche, die bei der Mehrheit der deutschen Bevölkerung vorliege und von den „Starken“ in der Bevölkerung kontrolliert werden müsste. Psychisch Kranke zählte er zu den Schwachen, die von der Gesellschaft isoliert werden müssten. Camerons psychiatrische Expertise und weltweite Anerkennung als Gründungspräsident der World Psychiatric Association (WPA) zusammen mit seiner missionarischen Überzeugung, die menschliche Gesellschaft vor krankhaften Ideen, insbesondere der angstbedingten Aggressivität der Deutschen schützen zu müssen, hat ihn offensichtlich dazu verführt, Ideen der sozialen Kontrolle zu entwickeln, die von denen der Nationalsozialisten kaum mehr zu unterscheiden waren.

So sah Cameron 1946 in einem Vortrag über „Frontiers of Social Psychiatry“ [36] die Aufgabe der Sozialpsychiatrie in einer grundlegenden Umgestaltung der sozialen Beziehungen. Denn nur Sozialpsychiater hätten die dafür erforderlichen Kenntnisse menschlichen Verhaltens, Fähigkeiten und Fertigkeiten. Er dachte sehr in sozialpsychologischen Mechanismen, insbesondere der pathogenen Weitergabe pathologischer Persönlichkeitszüge, sah pathogene Momente der Unsicherheit in Gruppen benachteiligter Menschen, forderte als „Sozial-Ingenieur“ soziale Kontrolle der „suitability for marriage“ und „quarantine of individuals suffering from diseases likely to spread to others“ [36]. Er benutzte Ausdrücke wie „Umwertung der (die Arbeit betreffenden) Werte“, die aus dem gleichen, kulturkritisch gespeisten sozialrevolutionären Impetus zu stammen scheinen, mit dem die Nationalsozialisten die „Umwertung aller Werte“^6^ gefordert hatten [36].

Camerons Tragödie war die „Kollision zwischen Wissenschaft und Menschlichkeit“: „Simply put, Cameron believed that he could better serve his patients by removing their memories and actualizing the theoretical concept of the tabula rasa or a ‘blank slate’ and building their personalities anew. He attempted to heal, but instead caused much hurt“ [38]. Die Tatsache, dass er die negativen Reaktionen seiner Patienten auf seine therapeutischen Maßnahmen explizit beschreibt, kann als Hinweis darauf verstanden werden, dass er völlig überzeugt davon war, das Richtige zu tun. Aber „Cameron … placed science (or, rather, his perception thereof) before the needs of his patients“. Anscheinend war er eher an der Menschheit als am einzelnen Menschen, stärker an seinen Konzeptionen als am individuellen Patienten interessiert. „It could also be argued that he was probably ‘too’ eager and relaxed scientific standards for the chance of a breakthrough success“ [38].

Cameron erscheint somit als ein Beispiel dafür, dass ein therapeutisch wie wissenschaftlich ungemein engagierter und organisatorisch befähigter Psychiater einerseits in praxi zu einem Initiator einer rehabilitativ orientierten Sozialpsychiatrie wurde, andererseits aber mit seiner auf ganze Bevölkerungen zielenden sozialtechnischen Ideologie in das Risiko lief, totalitäre Verformungen der Gesellschaft und die Exklusion psychisch Kranker zu fördern, ein Risiko, das sich bei dem ebenfalls therapeutisch sehr engagierten Carl Schneider furchtbar realisierte [39, 40].

## Footnotes

1 Ein Consentformular (Weinstein a. a. O. S. 32) aus dem Jahre 1956 war nichts anderes als eine Freistellung der Klinik und aller Mitarbeiter von jeglicher Haftung für potenzielle Schädigungen.

2 „It is the duty and the right of the physician to perform an experiment on man whenever it can save his life, cure him, or gain him some personal benefits.“ But Bernard also insisted on „never performing on man an experiment which might be harmful to him to any extent, even though the result might be highly advantageous to science“ zit. aus [24].

3 Nicht zuletzt unter diesem Eindruck wurde im folgenden Jahr die Allgemeine Deklaration der Menschenrechte von der UN verabschiedet; aber das Bewusstsein dafür, was sie in der konkreten Praxis bedeuten, entwickelt sich nur langsam.

4 Mit dieser Publikation setzte Henry Knowles Beecher eine Diskussion zur Ethik in der medizinischen Forschung in Gang, die sich in den USA zu einer grundlegenden bioethischen Bewegung entwickelte und damit den Kontext ärztlichen Handelns verwandelte: im Bulletin der WHO wurde Beechers Publikation charakterisiert als „the most influential single paper ever written about experimentation involving human subjects” and thereby “played a significant role” in enacting strict federal regulations [27].

5 Als Beispiel für das Dickicht der vielfältigen, gelegentlich auch emotional oder ideologisch beeinflusst wirkenden Publikationen mit ungenauen oder fehlerhaften Zitaten, unzutreffenden Quellenangaben oder geheim gehaltenen Quellen, sei nur die Kontroverse erwähnt zwischen dem Historiker McCoy A.W. [1] und den Psychologen Brown R [41] und Blass T [42].

6 unter Bezug auf einen Begriff des Philosophen Friedrich Nietzsche.
